# A challenging case of Mesenchymal Chondrosarcoma involving the thyroid and special considerations for diagnosis

**DOI:** 10.1186/s40842-020-00094-4

**Published:** 2020-03-11

**Authors:** Noura Nachawi, Madelyn Lew, Kristine Konopka, Zahrae Sandouk

**Affiliations:** 1 Department of Internal Medicine, Saint Joseph Mercy Hospital Ann Arbor, Ann Arbor, MI USA; 2 Department of Pathology, University of Michigan, Ann Arbor, MI USA; 3 Department of Internal Medicine, Division of Metabolism, Endocrinology and Diabetes, University of Michigan, Ann Arbor, MI USA

**Keywords:** Thyroid nodule, Mesenchymal chondrosarcoma, Fine-needle aspiration, Core-needle biopsy

## Abstract

**Background:**

Thyroid ultrasound is usually used to risk-stratify incidental thyroid nodules. Nodules with high risk sonographic features for malignancy are evaluated by fine-needle aspiration. The role of core needle biopsy for thyroid nodules is limited to cases where the fine needle aspiration is inconclusive.

**Case presentation:**

We describe a rare case of mesenchymal chondrosarcoma of the thyroid gland with uncertain primary origin. Thyroid ultrasound showed right sided large, solid, hypoechoic nodule with calcifications and peripheral vascularity and unremarkable isthmus and left thyroid lobe. Fine needle aspiration of the right nodule suggested lymphocytic thyroiditis. The sonographic findings contradicted the typical bilateral clinical and sonographic picture of lymphocytic thyroiditis. A core needle biopsy showed mesenchymal chondrosarcoma.

**Conclusion:**

This case highlights the importance of correlating pathologic diagnosis with sonographic findings, the appropriate utilization of fine needle aspiration and core needle biopsy to evaluate thyroid nodules and the rare incidence of mesenchymal chondrosarcoma involving the thyroid.

## Background

Thyroid nodules are commonly discovered during physical exam or incidentally found on imaging done for other purposes. Most thyroid nodules are benign. Thyroid cancer occurs in 5–14% of thyroid nodules [1, 2]. Rare thyroid cancers including undifferentiated and medullary, thyroid cancer account for less than 3% of all cases.

Differentiation between malignant and benign thyroid nodules starts with their sonographic characteristics. In 2015, the American Thyroid Association published guidelines on the appropriate approach and management of thyroid nodules [3]. Per these guidelines, fine-needle aspiration (FNA) biopsy is indicated in any non-functional thyroid nodule measuring > 1 cm (cm) in greatest diameter with intermediate and/or high suspicion (70–90%) for malignancy. Some studies suggest that core-needle biopsy of thyroid nodules demonstrates high rates of conclusive and accurate diagnoses in patients for whom previous fine-needle aspiration results were non-diagnostic [4].

Both primary and metastatic mesenchymal chondrosarcoma found in the thyroid gland are extremely rare. Twenty percent of these tumors have metastatic disease at diagnosis [5]. Reported 10-year survival rates range from 10 to 54% [5–10].

In this case, we report a rare presentation of mesenchymal chondrosarcoma of the thyroid gland. We also illustrate the role of core needle biopsy to diagnose highly suspicious thyroid nodules when the fine needle aspiration is inconclusive.

## Case report

A 24-year-old Asian man, with remote history of lung lesion removal in his home country as a child (< 10 years of age), was found to have enlarged right thyroid lobe on exam by his primary care physician. He did not know more details about his remote diagnosis and no medical records were available. He had no past history of radiation exposure. No symptoms of thyrotoxicosis, hypothyroidism or compressive thyromegaly were reported. Family history was unremarkable for any thyroid related diseases or malignancies. He was not on any medications. Review of systems was unremarkable. He denied having cough, dyspnea, fatigue. Examination was unremarkable except for a large soft, non-tender, mobile nodule on the right thyroid, but no palpable lesions on the left side. No noted lymphadenopathy. His labs were unremarkable with a TSH level of 2.51 mIU/L and undetectable microsomal antibody level of < 10 IU/mL.

**Fig. 1:**
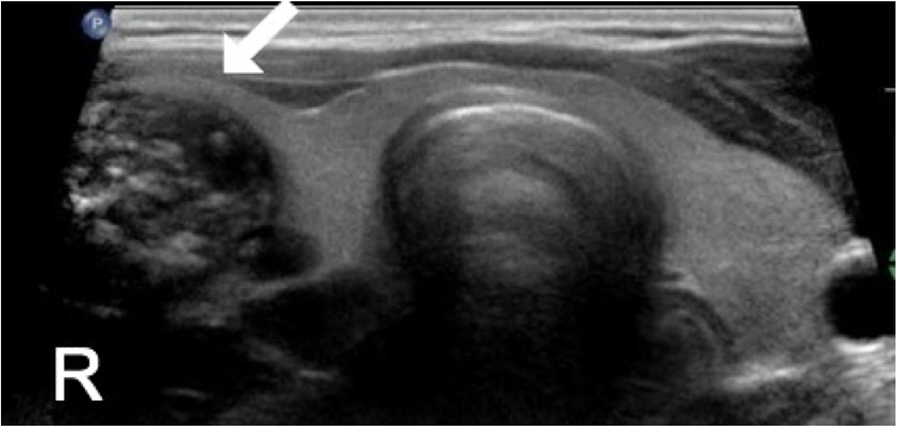
Second ultrasound. Transverse view of the thyroid ultrasound showing right lobe, isthmus and left lobe. The isthmus and the left lobe are noted for homogenous echotexture. Right lobe (white arrow) is enlarged due to multiple nodules

**Fig. 2:**
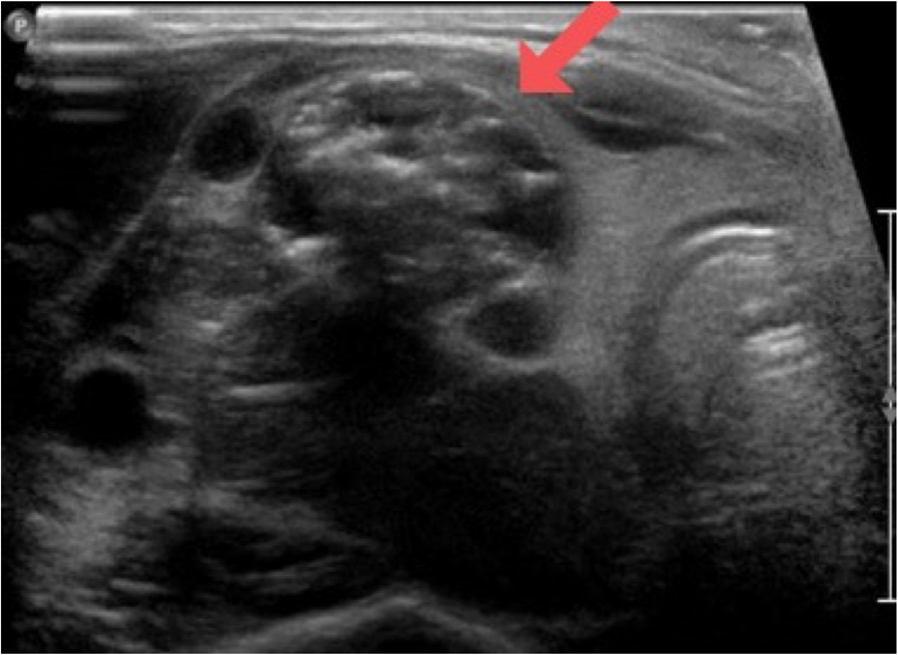
Second ultrasound. Transverse view thyroid ultrasound showing the enlarged right thyroid lobe consisting of multiple solid hypoechoic nodules some with calcifications. The largest nodule in right mid-pole (red arrow) measures 2 × 1.6 × 2.1 cm

A thyroid ultrasound, showed enlarged right thyroid with multiple solid hypoechoic nodules some with coarse calcifications, largest in right mid-pole measuring 2 × 1.6 × 2.1 cm with peripheral vascularity. The left thyroid lobe and isthmus were both noted for homogenous echotexture and normal vascularity. (Figures 1 and 2). Given the size and the sonographic pattern of the nodules, a fine-needle aspiration biopsy was performed. Cytology results were consistent with Bethesda II, benign, with features of lymphocytic thyroiditis. However, lymphocytic thyroiditis - also known as Hashimoto’s disease - usually involves both thyroid lobes, which was not the case in our patient. So a repeat ultrasound was done at 6 months and endocrine referral was placed by his primary care physician.

**Fig. 3:**
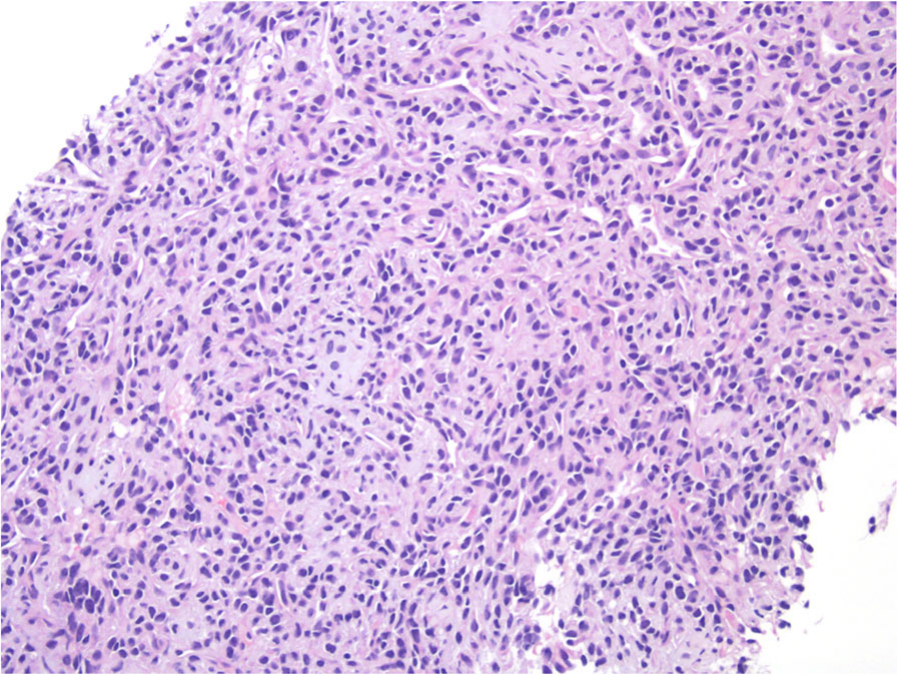
The core biopsy material shows a population of malignant cells characterized by round to ovoid and hyperchromatic nuclei around slit-like to staghorn-shaped vascular spaces, consistent with mesenchymal chondrosarcoma. Hematoxylin & Eosin, 20x magnification

Repeat ultrasound evaluated by endocrinology showed a stable right sided nodule, measuring 1.6 × 2.0 × 2.2 cm with smooth margins, hypoechoic with increased macro and microcalcifications. Core-needle biopsy was recommended and revealed mesenchymal chondrosarcoma with preliminary grade of 2 out of 3 on the French Federation of Cancer Centers Sarcoma Group (FNCLCC) (Fig. 3).

**Fig. 4:**
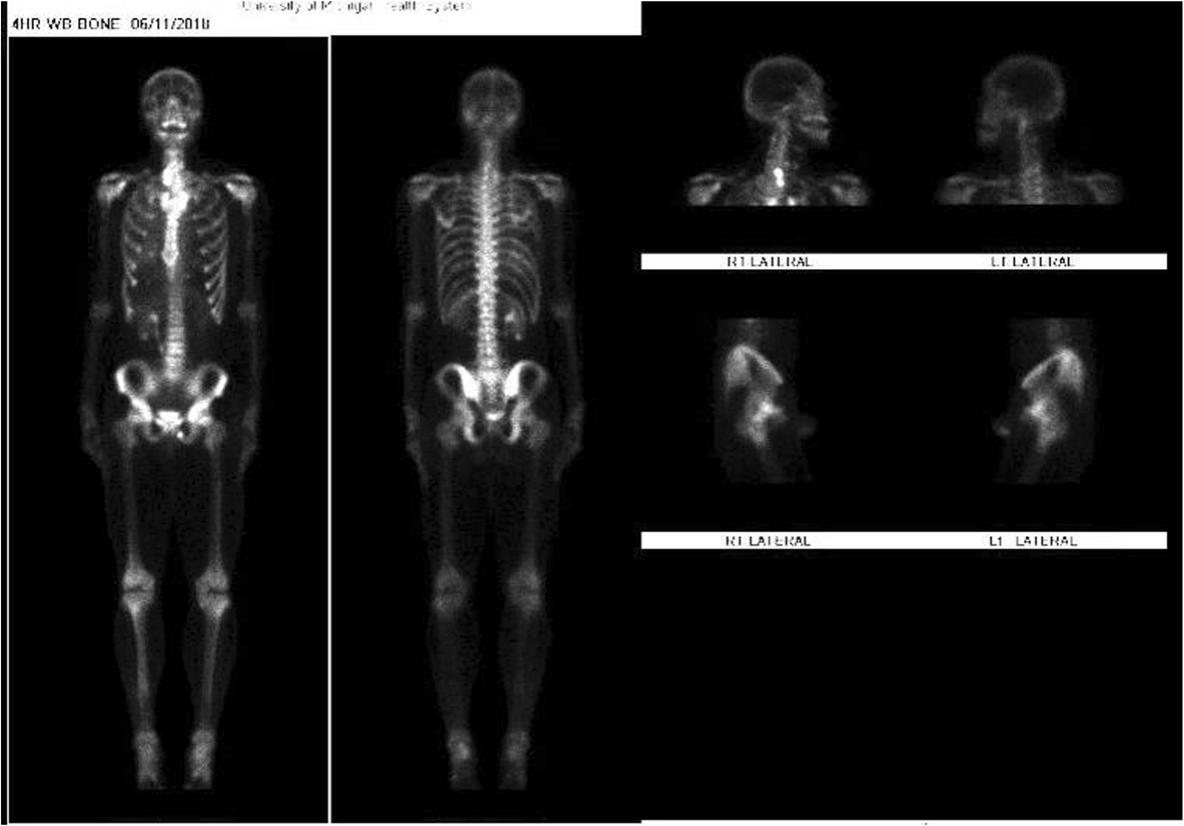
Bone Scan with SPECT/T showing lesions involving the thyroid, lung, right pleural, and right scapula

This prompted an immediate referral to Oncology, sarcoma specialist who recommended further imaging - staging was performed over the course of 3 weeks. Computed Tomography (CT) of the neck re-demonstrated large right thyroid mass with calcified matrix. CT of the chest noted innumerable nodules concerning for metastatic disease and loculated right pleural effusion with cellular debris versus pleural deposits. Bone scan with SPECT-CT showed the known heterogeneous soft tissue attenuation right thyroid mass with two areas of dense calcifications (Fig. 4). There was a large amount of dense soft tissue attenuation throughout the right pleura without abnormal radiotracer uptake. Post-surgical changes consistent with partial right 7th rib resection were also identified, presumably form the patient’s prior surgical intervention. There was also focally increased radiotracer uptake along the superior aspect of the right scapular spine, with corresponding lytic lesion, concerning for bone metastatic lesion. Ultimately, imaging studies concluded the final findings of mesenchymal chondrosarcoma of unclear primary involving the thyroid, lung, right pleural, and right scapula.

During that period of time, patient reported worsening dry cough, nocturnal shortness of breath, intermittent neck and back discomfort and profound fatigue. His fatigue limited his ability to cook for himself and he had lost almost 10 pounds. Upon follow up with sarcoma oncology, the metastatic mesenchymal chondrosarcoma was deemed non-curable but chemotherapy options were given for tumor control. The main goals and regimens of chemotherapy were discussed with the patient. Patient decided to go back to his home country to receive treatment where his family can closely support him.

## Discussion

This case highlights several interesting points. Based on the ultrasound characteristics, the nodule fell in the high suspicion risk category due to presence of microcalcifications and hypoechoic component of a partially cystic nodule. The American Thyroid Association 2015 guidelines recommends fine-needle aspiration biopsy for all nodules larger than 1 cm in greatest dimension with high suspicion sonographic pattern (Strong recommendation, Moderate-quality evidence) [3]. Suspicious features for thyroid cancer include the presence of microcalcifications, nodule hypoechogenicity compared with the surrounding thyroid or strap muscles, irregular margins and a shape taller than wide measured on a transverse view. Features with the highest specificities for thyroid cancer are microcalcifications, irregular margins, and tall shape [3]. Up to 55% of benign nodules are hypoechoic compared to thyroid parenchyma, making nodule hypoechogenicity less specific. The risk of malignancy is higher for hypoechoic nodules with microcalcifications than for hypoechoic solid nodules lacking these features [11]. In our patient, the largest thyroid nodule was described as hypoechoic with a mix of micro and macro calcifications. It measured more than 2 cm. All of these features suggest a 70–90% risk for thyroid malignancy.

Furthermore, the patient’s left thyroid lobe and isthmus were noted to be homogenous and unremarkable on ultrasound. Hashimoto’s disease typically manifests as diffusely hypoechoic bilateral thyroid parenchyma, heterogeneous echotexture and the presence of micronodules on ultrasound. Lymphocyte infiltration, typically involving both lobes, underlie the main etiology of decreased echogenicity seen on ultrasound [12–15]. Our patient’s benign histopathologic findings from initial fine needle aspiration were discrepant with his high risk sonographic features. Endocrinology consultation 6 months later included core needle biopsy, revealing grade 2 mesenchymal chondrosarcoma. It is uncertain whether doing core-needle biopsy on the initial presentation would have resulted in earlier diagnosis of the aggressive mesenchymal chondrosarcoma. Many studies have compared fine needle aspiration with core needle biopsy in the evaluation of mesenchymal tumors. One case series have looked into 377 thyroid biopsies done with both fine needle aspiration and core-needle biopsy [16]. In that study, fine-needle aspiration was performed with either concurrent or subsequent core needle biopsy. 62 patients (16.4%) underwent subsequent surgical resection, yielding 32 malignancies in 31 patients (28 primary thyroid malignancies, 2 lymphomas, and 2 metastatic renal cell carcinomas). The study concluded that when the two biopsy modalities were combined, there were no false-positive or false-negative diagnoses of thyroid malignancies. However, it was noted that not every patient with negative biopsy results had surgical follow-up for histologic confirmation. The adequacy rate for core-needle biopsy was significantly higher than that of FNA for evaluation of primary thyroid malignancies, but the combined adequacy was significantly higher than that for either test alone. Complications of core needle biopsy included increased bleeding and hematoma not requiring hospitalization [16]. Although this study did not include any mesenchymal malignancies in its positive cohort, it emphasizes the beneficial use of core needle biopsy when fine needle aspiration of high risk thyroid nodule is undiagnostic. This was also reported in Na DG et al. 2012 [17] who concluded that core needle biopsy is more useful than repeat FNA for reducing the frequency of inconclusive diagnostic results (Bethesda I and III). This approach may reduce false negative results and expedite the diagnosis of cancerous thyroid lesions. Additionally, literature review supports utilizing core needle biopsy particularly to diagnose malignancies of musculoskeletal origin. One retrospective study on 57 patients with extremity soft tissue palpable masses found that core needle biopsy has higher specificity and accuracy when compared to fine-needle aspiration [18]. Similarly, a meta-analysis suggested that core needle biopsy should be performed to diagnose soft tissue and bone sarcomas prior to more invasive surgical biopsy [19].

Mesenchymal chondrosarcomas are highly malignant. They usually comprise differentiated cartilage admixed with solid highly cellular areas consisting of undifferentiated small round cells [20]. Up to one third of mesenchymal chondrosarcoma primarily originates from extra-skeletal soft tissues and can also involve axial bones. Twenty percent of cases are metastatic on initial diagnosis [5]. They have a tendency toward both local and distant recurrences, which may arise as long as 20 years following the initial diagnosis [8]. Retrospectively, earlier repeated ultrasound and biopsy for our patient would have been helpful in earlier diagnosis.

Our patient presentation remains extraordinarily rare despite the uncertainty of the primary origin of mesenchymal chondrosarcoma in his case. Both primary and secondary involvement of the thyroid by mesenchymal chondrosarcoma are astonishingly uncommon with only one primary case reported in the literature [21]. Additionally, only a handful of previous cases of chondrosarcoma metastasis to the thyroid have been reported. Only one case of the mesenchymal chondrosarcoma subtype metastasizing to the thyroid gland was reported in the literature [22]. Both of these cases were different from our case.

In the first case reporting mesenchymal chondrosarcoma of the thyroid, the patient age, clinical course and sonographic features were different from the case we describe here [21]. In that case, the patient was a 13-year-old female who presented with thyroid swelling without compressive symptoms. Thyroid ultrasound showed a 1.8 X1.5 X 1.2 cm well-defined hyperechoic nodule with multiple internal echoes, consistent with a colloid cyst of the right lobe of the thyroid. Fine-needle aspiration revealed cellular smears containing dispersed and clustered cells, with oval or spindle-shaped nuclei and no atypia. Patient’s guardian declined resection for histological diagnosis. She presented 2 years later with worsening swelling. Fine-needle aspiration showed features consistent with a follicular neoplasm with an excessive stromal reaction. Neck exploration followed by right hemithyroidectomy was performed. A final diagnosis of a mesenchymal chondrosarcoma was made. Further workup revealed no metastasis or recurrence of the disease at 66 months from initial presentation.

The first case reporting metastatic mesenchymal chondrosarcoma to the thyroid also had a different presentation from our case [22]. In that case, a 23-year-old woman was diagnosed with mesenchymal chondrosarcoma of the sacrum. Complete CT scan of the body did not reveal any distant metastasis at the initial diagnosis. She underwent total resection of multiple sacral vertebrae with reconstruction of the pelvis and the diagnosis of mesenchymal chondrosarcoma was confirmed. Surgery was followed by chemotherapy. Three years following initial presentation, patient developed painless swelling of the thyroid gland. A computed tomography (CT) scan showed a well-defined, heterogeneous and bulky tumor of the right thyroid lobe, with a diameter of about 4 cm. Fine needle aspiration cytology revealed atypical cells and a subsequent core biopsy disclosed a lesion compatible with a metastatic malignancy. Right lobectomy was performed and the final pathology report confirmed the metastatic mesenchymal chondrosarcoma.

## Conclusion

In conclusion, our case highlights the importance of recognizing thyroid nodules with highly suspicious ultrasound features of malignancy. It is substantially significant to review the biopsy results in the context of the sonographic and clinical features. Core needle biopsy should be considered to aid further diagnosis whenever the fine-needle aspiration results are not convincing, particularly if there is a history of clinical concern for a mesenchymal lesion, which can be more difficult to aspirate cells from and to characterize accurately on cytology. Moreover, further studies and future guidelines are needed to guide healthcare providers managing similar cases with equivocal fine-needle aspiration results. Mesenchymal chondrosarcoma primarily arising from the thyroid gland or metastasizing to the thyroid gland is extremely rare. Given their tendency for early and late recurrence and poor prognosis, early diagnosis is highly stressed to avoid delayed diagnosis.

## Data Availability

The data used in this case report are available in the patient’s medical record and can be disclosed by the corresponding author on reasonable request.

## Abbreviations

cm: Centimeter
CT: Computerized tomography
FNA: Fine-needle aspiration
FNCLCC: Fédération Nationale des Centres de Lutte Contre le Cancer
IU/mL: International unit per milliliter
mIU/L: Milli international unit per liter
SPECT: Single Photon Emission Computed Tomography
